# Subcellular RNA Sequencing Reveals Broad Presence of Cytoplasmic Intron-Sequence Retaining Transcripts in Mouse and Rat Neurons

**DOI:** 10.1371/journal.pone.0076194

**Published:** 2013-10-03

**Authors:** Mugdha Khaladkar, Peter T. Buckley, Miler T. Lee, Chantal Francis, Mitra M. Eghbal, Tina Chuong, Sangita Suresh, Bernhard Kuhn, James Eberwine, Junhyong Kim

**Affiliations:** 1 Department of Biology, University of Pennsylvania, Philadelphia, Pennsylvania, United States of America; 2 Penn Genome Frontiers Institute, University of Pennsylvania, Philadelphia, Pennsylvania, United States of America; 3 Department of Pharmacology, University of Pennsylvania, Philadelphia, Pennsylvania, United States of America; 4 Genomics and Computational Biology Program, University of Pennsylvania, Philadelphia, Pennsylvania, United States of America; 5 Department of Genetics, Yale University, New Haven, Connecticut, United States of America; 6 Department of Pediatrics, Boston Children’s Hospital, Boston, Massachusetts, United States of America; Yale School of Medicine, United States of America

## Abstract

Recent findings have revealed the complexity of the transcriptional landscape in mammalian cells. One recently described class of novel transcripts are the Cytoplasmic Intron-sequence Retaining Transcripts (CIRTs), hypothesized to confer post-transcriptional regulatory function. For instance, the neuronal CIRT KCNMA1i16 contributes to the firing properties of hippocampal neurons. Intronic sub-sequence retention within IL1-β mRNA in anucleate platelets has been implicated in activity-dependent splicing and translation. In a recent study, we showed CIRTs harbor functional SINE ID elements which are hypothesized to mediate dendritic localization in neurons. Based on these studies and others, we hypothesized that CIRTs may be present in a broad set of transcripts and comprise novel signals for post-transcriptional regulation. We carried out a transcriptome-wide survey of CIRTs by sequencing micro-dissected subcellular RNA fractions. We sequenced two batches of 150-300 individually dissected dendrites from primary cultures of hippocampal neurons in rat and three batches from mouse hippocampal neurons. After statistical processing to minimize artifacts, we found a broad prevalence of CIRTs in the neurons in both species (44-60% of the expressed transcripts). The sequence patterns, including stereotypical length, biased inclusion of specific introns, and intron-intron junctions, suggested CIRT-specific nuclear processing. Our analysis also suggested that these cytoplasmic intron-sequence retaining transcripts may serve as a primary transcript for ncRNAs. Our results show that retaining intronic sequences is not isolated to a few loci but may be a genome-wide phenomenon for embedding functional signals within certain mRNA. The results hypothesize a novel source of cis-sequences for post-transcriptional regulation. Our results hypothesize two potentially novel splicing pathways: one, within the nucleus for CIRT biogenesis; and another, within the cytoplasm for removing CIRT sequences before translation. We also speculate that release of CIRT sequences prior to translation may form RNA-based signals within the cell potentially comprising a novel class of signaling pathways.

## Introduction

Recently, several studies have revealed a new dimension to the diversity of transcripts [1], for instance thousands of circular RNAs many of which reside within introns as reported in [2] and the extensive presence of chimeric transcripts with coordinated expression of connected genes [3]. Many researchers have historically reported findings of intronic sub-sequences in expressed transcripts [4,5]. These “retained intronic” sub-sequences have been previously attributed to cryptic exons or mis-splicing events [6-9]. But, more recently, several papers have suggested that ***non-coding*** retained intronic sub-sequences may play a specific functional role in post-transcriptional regulation. Post-transcriptional regulatory function arises through the retention of specific intronic, nonprotein-coding sequences within the cytoplasmic mRNA. We call such sequences Cytoplasmic Intron-sequence Retaining Transcripts (CIRTs). For instance, the neuronal CIRT KCNMA1i16 contributes to the firing properties of hippocampal neurons and proper channel protein localization to dendrites [10]. Intron sub-sequence retention within IL1-β mRNA in anucleate platelets has been implicated in governing activity-dependent splicing and translation upon cell activation [11]. A retained intronic region in Tap mRNA contains a transport element that drives nuclear export in human 293T cells, facilitating expression of an alternate Tap protein product [12]. In a recent study, a set of 33 candidate genes with mRNA previously found to localize in dendrites of rat were shown to contain many CIRTs that also harbor functional SINE ID elements which are hypothesized to mediate dendritic localization in neuronal transcripts [13]. These studies raises the question of how extensive is the intron-sequence presence in the transcriptomes of mammalian cells.

Here we performed a transcriptome-wide survey for intronic sequence retention using subcellular RNA sequencing of dissected neurites from rat and mouse hippocampal neurons. Neurons are highly polarized cells with spatially distinct dendrites and axons that allow mechanical isolation of cytoplasmic RNA through microdissection. Strictly speaking, our subcellular dissections do not distinguish dendrites and axons but in these neurons the axonal compartment is less than 5% of the volume of dendritic compartments; therefore, we will call our samples dendritic samples from hereon. In addition to cytoplasmic RNA from dendrites, for contrast we also carried out single cell RNA sequencing of individual cell somas, which also contains some heterogeneous nuclear RNA (hnRNA). These datasets were augmented by single cell sequencing from mouse Brown adipose tissue (BAT) [14] and mouse cardiomyocytes (see Methods). After having filtered for other possible origins for these intronic reads in RNA-seq data we found evidence for CIRT variants in 44% of rat dendritic genes and in 59% of mouse dendritic genes. In addition, we found evidence for CIRTs in ~28% of genes in rat neuronal soma samples and ~59% of genes in mouse neuronal soma samples. By clustering the sequence reads mapping to each intron we found that the average length of the retained intronic region was ~ 360 bp in the rat dendrites and ~353 bp in mouse dendrites. In addition, we also report here evidence for several novel intron-intron junctions. Single cell samples from BAT and cardiomyocytes also revealed high level of intronic sequences in their single cell transcriptome (~61% of genes in BAT cells and ~49% in cardiomyocytes). Our findings of widespread prevalence of CIRTs in all of the cell types investigated further emphasizes the hitherto unknown transcript diversity. 

## Results

### Widespread evidence of CIRTs within dendritic mRNAs of rodent neurons

We performed a comprehensive transcriptome wide survey of the prevalence of CIRTs in rat and mouse dendritic population. Two batches of dendritic mRNA from primary rat hippocampal neurons and three batches of dendritic mRNA from primary mouse hippocampal neurons, each consisting of 150–300 individually dissected dendrites were independently aRNA amplified [15] and subjected to Illumina NextGen sequencing. 

Of the reads mapping to intronic regions, only the uniquely mappable reads by paired-end Bowtie alignment [16] were counted. Mechanically dissected dendrites are expected to contain only non-nuclear RNA (see 13). However, to overcome the potential issue of nuclear hnRNA contamination as well as the presence of cryptic transcriptional units (e.g., cryptic exons, anti-sense RNA, etc), we carried out computational filtering to obtain the most probable set of cytoplasmic intron sequences. 

First, each intron with putative cytoplasmically retained sequence was also filtered for overlap with predicted genes using the tools GENSCAN [17] and TWINSCAN [18] to minimize any other possible cryptic translated sequences. Second, we also screened the introns with retained sequence for the presence of intronic poly(A)-sites by using the PolyA_DB2 database which reports poly(A) sites obtained using alignments between cDNA/ESTs and genome sequences [19], and removed those that contained a poly(A) site. Third, we examined the remaining reads for pattern of sequence-read coverage and found no significant enrichment in intergenic reads (Binomial proportion test P-value < 0.01 for enrichment in intronic reads as compared to intergenic and P-value < 2e-16 for enrichment in exonic reads as compared to intergenic reads, also see 13), nor did we find any systematic pattern of directional bias in intron presence or absence across all genes that might occur with partial products of nuclear splicing. Forth, we analyzed the possibility that independently transcribed ncRNAs may be present within the introns that give rise to the CIRTs. *Guttman et al* [20] reported large noncoding RNAs in mouse ESC, NPC and MLF. Here we compared the genomic coordinates of the noncoding RNAs found by the study to the genomic coordinates of the retained intronic contigs, and to be conservative even a single base overlap between the two coordinates was considered to be a potential ncRNA. Only 30 of the 30,124 introns with retained sequence from mouse samples overlapped with the putative ncRNAs identified by this study. We also analyzed all RNA families and RNA genes from mouse and rat that have been annotated in Rfam [21] for overlap with the introns with retained sequence. Less than 2% of the retained introns in mouse and rat samples overlapped with Rfam annotated RNA families and RNA genes. Fifth, another possible origin of the CIRTs is from non-specific anti-sense transcription [22]. Since the RNA-seq protocol that we followed is not strand specific, it is difficult to completely rule out the possibility of antisense transcription resulting in these intronic reads. However, to get a lower limit on the prevalence of antisense transcription in the regions overlapping with the retained introns that we have reported here, we made use of the multi-exonic antisense transcripts from mouse embryonic stem cells (ESC), neural progenitor cells (NPC) and mouse lung fibroblasts (MLF) reported by a recent study that carried out *ab inito* reconstruction of these transcriptomes [20]. We found that <5% of the retained introns from mouse samples have at least a partial overlap with these antisense transcripts. Thus, although we cannot completely rule out all instances of antisense transcription, this comparison suggests that a majority of the retained introns are not likely to be a result of antisense transcription. Finally, a small fraction of the reads mapped to the exon-intron junctions and were the only reads that mapped to the particular intron. It is possible that these reads represent an extension of the exonic sequence and correspond to un-annotated alternate exon start or end sites. Hence, we also excluded these introns from all further analysis. 

After processing through these steps to minimize other origins of intronic sequences in the mRNA sequences, we found ~44% rat genes and ~59% of the mouse genes in dendritic samples showing intron sequence read evidence consistent with the hypothesis of CIRTs (Table 1, Binomial test p-value < 2.2e-16). For these genes, the read density in the exonic region is poorly correlated with the read density of the intronic region (r = 0.01, P-value = 0.2, Sample size = 4485) which suggests that the CIRTs are one of the isoforms of the gene. The median read density in the intronic regions of these genes, albeit not uniform within any given intron, was 3 reads per 50 bp for rat dendrites and 7 reads per 50 bp for mouse dendrites while the maximum read density was 950 reads per 50 bp for rat dendrites and 2350 reads per 50 bp for mouse dendrites. We also identified a set of transcripts in dendritic samples of rat and mouse wherein the sequence evidence comes only from within the intronic regions of a gene model (7-14%) (Table 1). For these transcripts the median read density was 1 read per 50 bp for rat dendrites and 4 reads per 50 bp for mouse dendrites and the maximum was 290 reads per 50 bp for rat dendrites and 950 reads per 50 bp for mouse dendrites. We analyzed the mRNA half life for these mouse transcripts to see whether they have a lower half life than the rest of the transcripts which may explain the lack of exonic reads; the intronic reads may correspond to those transcripts that exist for a longer duration. We used a published database of mRNA decay rates in pluripotent and differentiating mouse embryonic stem cells [23] and did not find any difference in the decay rates of the intron-only transcripts as compared to the transcript with both exonic and intronic reads. We have reported previously [13,24] that considerable biological transcriptome variation exists at the single cell level. Thus, we expected a distinct set of CIRTs for different samples. For the biological replicates, the overlap in the identity of the genes that gave rise to CIRTs was ~33% on average. For these shared genes the overlap in the retained introns was ~ 23% on average. A small fraction of the genes give rise to potential CIRT evidence with the same intron for all sequenced samples (648 genes (7%) for mouse dendrites and 687 genes (15%) for rat dendrites), which we consider to be the most confident CIRT set (Table 1). We analyzed the fraction of transcript models showing intron sequence read evidence consistent with the hypothesis of CIRTs in the sequenced dataset as a function of expression levels. The fraction of transcript models with potential CIRT evidence drops roughly linearly as a function of log expression (Figure S1 in File S1). A cutoff of 32 reads from at least one of the retained introns in any transcript resulted in ~21% of the rat genes and ~37% of mouse genes from dendritic samples showing evidence of CIRTs. Even with a very high cutoff of 256 reads ~7% of rat genes and ~14% of mouse genes from dendritic samples showed evidence of CIRTs. Hence we conclude that CIRTs are a widespread presence in the neuronal dendritic population.

**Table 1:** Prevalence of CIRTs in dendritic and soma mRNAs from rat and mouse hippocampus. neurons.

**Sample**	**Total Genes**	**Genes with exonic reads**	**Genes with exonic and intronic reads**	**Genes with only intronic reads**	**Total Genes with intronic reads**	**Genes with a given intron retained in all samples**
**Mouse/Dendrite**	14,478	12,344	6,473 (52.4%)	2,134 (14.7%)	8,607 (59.4%)	648 (7.5%)
**Rat/Dendrite**	10,104	9,308	3,689	796	4,485	687
			(39.6%)	(7.8%)	(44.4%)	(15.3%)
**Rat/Soma**	8,750	8,308	2,079	442	2,521	110
			(25%)	(5.1%)	(28.8%)	(4.4%)
**Mouse/Soma**	12,200	11,255	3,990	945	4,935	115
			(35.4%)	(7.7%)	(40.4%)	(2.3%)

### Cytoplasmic retention of intronic regions is limited to a small fraction of introns within the gene and sequence evidence arises from a short region within these introns

**Figure 1:**
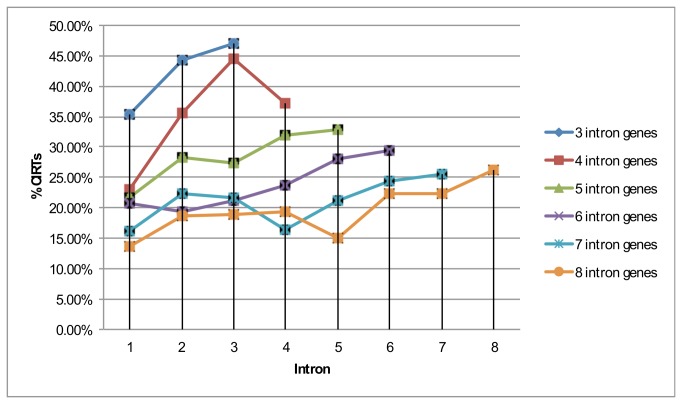
Frequency of CIRTs as a function of 5’ to 3’ intron positions of the gene model.

To estimate the length frame of the retained intronic sequence, we clustered the reads that were separated by no more than 500 bp to form contiguous regions, which we call contigs (Experimental Procedures). We chose to have this larger distance threshold in order to account for the reads arising from repetitive sequences that tend to be enriched in intronic regions and may not be uniquely mappable and also to be conservative in the number of independent putative CIRTs. This distance is also roughly equal to two back-to-back paired-end reads. Of all the possible introns within the genes expressed, we found that cytoplasmic retention occurs in only a small fraction of the introns (13-16%) (Table 2) in rat as well as in mouse dendritic samples. The average fraction of retained introns per gene amongst all introns of that gene was 1. The fraction of retained introns per gene showed very low correlation with read depths (rank correlation r=0.03, p-value = 0.009; significant but small effect size, n = 4485) suggesting that the detection of a restricted subset of possible introns is not due to lack of read coverage (Figure S2 in File S1). Thus, the putative cytoplasmically retained intronic reads arise from a specific subset of introns rather than through random retention of intronic sequences. A large fraction of these introns gave rise to a single contig; on average 86% of all retained intronic sequences are within a single contig. Since the mRNA amplification protocol recognizes the poly-A tail for amplification [25], there is a possibility for a bias towards 3’-most introns in the detection of intronic retention. We analyzed whether the set of retained introns occurs primarily from the 3’ half of the transcript. The results showed a moderate significant difference of ~7% in the proportion of retained intronic sequences in the 5’ half and 3’ half of all the genes with >= 5 introns combined (Table S1 in File S1), but reversed in direction between mouse and rats, indicating the difference is not likely due to the amplification protocol which is the same for both species. There was no pattern of 5’ or 3’ bias of introns in the CIRTs and the frequency of CIRTs was uniform as a function of 5’ to 3’ introns of a gene model (Figure 1). We also found that ~47% of all retained introns contained an internal pA tract of length >= 10 bp (Table S2 in File S1). It is likely that these pA tracts are used during mRNA amplification and thus they allow us to sample intronic sequences distal from the poly-A end of a transcript. We scanned these contigs for the maximal open reading frame (ORF) length in all six frames (three in the sense and three in the antisense orientation) and found a large majority of the contigs (>75%) to have an ORF length of less than 60 amino acids. Also, >80% of the retained intronic contigs have at least one stop codon in all six frames in the sense and antisense orientation. Therefore, the possibility that these contigs code for proteins is unlikely. These contigs do not appear to have a preference for a specific region of the intron. We divided the intron into three equal parts and computed the fraction of contigs falling within each part. The fraction of contigs lying in the beginning one-third of the intron appears to be higher for intron sizes upto 20,000 nt but the trend is largely random beyond that (Figure S3 in File S1). Also, ~30% of the retained intronic contigs show evidence for intron-exon junction reads at one of its ends. This suggests that at least some of these retained intronic contigs are in-frame retained with other exons and part of the spliced mRNA.

**Table 2:** Fraction of introns that are retained in CIRTs.

**Sample**	**# Introns in transcripts with intronic retention**	**# Retained introns **	**# Retained introns shared by all biological replicates**	**#Contigs in the retained introns**
**Mouse/Dendrite**	117,236	19,477 (16.6%)	773	26,445
**Rat/Dendrite**	56,932	7,426 (13.1%)	815	8,459
**Rat/Soma**	34,186	3,499 (10.2%)	119	3,784
**Mouse/Soma**	73,598	7,660 (10.4%)	122	8,691

**Figure 2:**
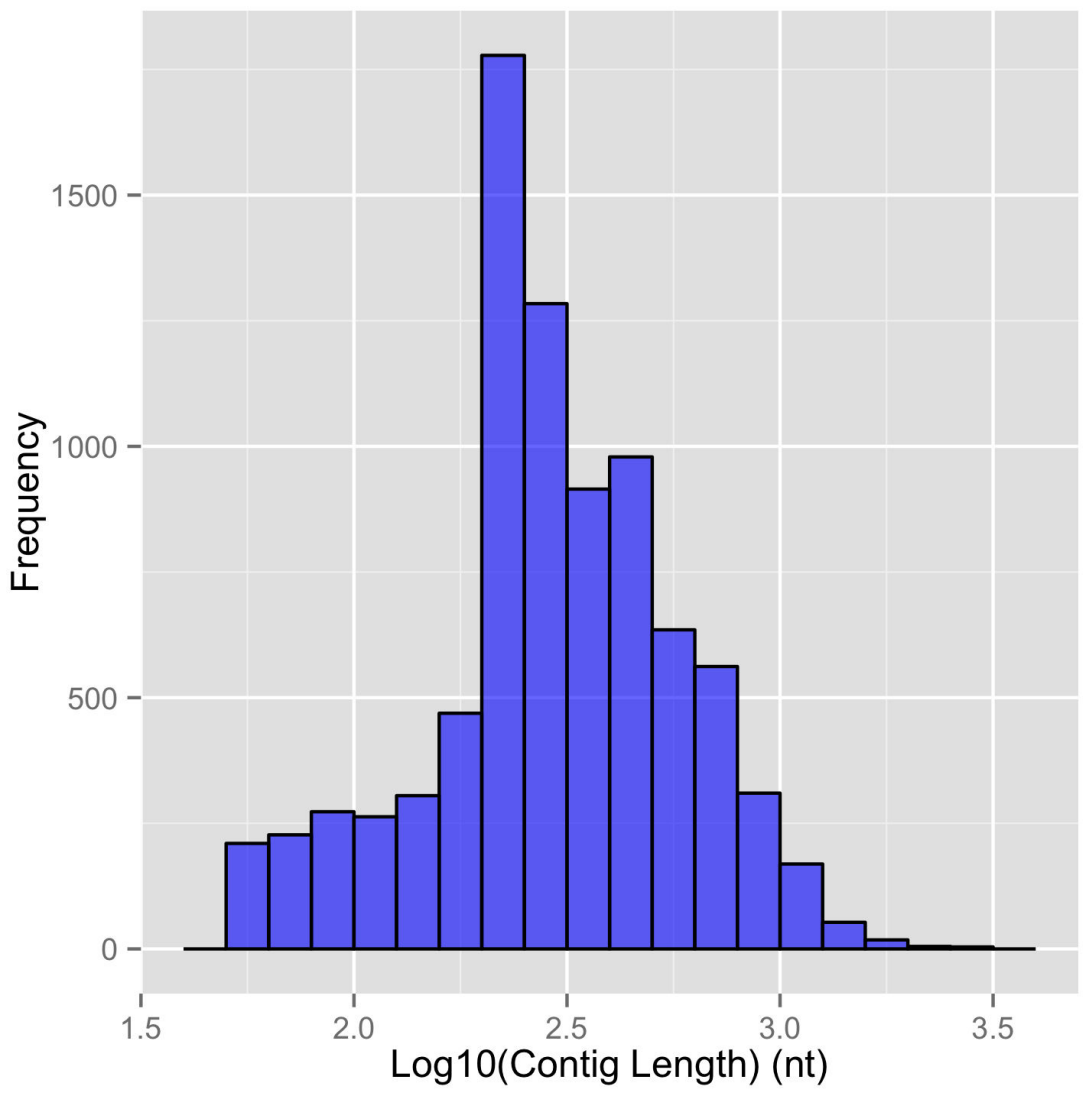
Length distribution for the intronic contigs in Rat dendrites.

The retained intronic contigs in rat dendritic samples had a mean length of 363 nt while in mouse dendritic samples the mean length was ~353 nt (Figure 2). The mean intron length in rat and mouse is ~4800 nt and ~5000 nt respectively. Thus we observe that on average a short segment of the introns is cytoplasmically retained. The distribution for the contig lengths of dendritic CIRTs deviates from an exponential distribution that would be expected if the sequenced intervals were generated from uniformly chosen random breaks in the sequences, suggesting that the length of retained intronic sequences within CIRTs is not due to random starts and ends (Kolmogorov-Smirnov test P-value < 2.2e-16). 

The aRNA amplification protocol uses poly(T)-T7 primers which recognizes pA-tracts including those from intronic regions. To assess whether the contig length distribution is related to chance occurrence of pA tracts within introns, we first constructed contigs of pA tracts by clustering pA sequences at least 9 bp long and separated by less than 500 bp. Of all the introns with cytoplasmically retained sequence in rat dendritic samples, ~56% had at least one internal pA tract. For these introns, there appeared to be no correlation between the average pA tract contig length and the average intronic contig length (*r* = 0.001, P-value = 0.9, Sample size = 3947). Another factor that may affect the resulting length of the contigs is the multiple rounds of aRNA amplification steps in our protocol, which can cause a shortening of the transcripts due to random primer binding for second-strand synthesis in each subsequent cycle. However, in rat dendritic samples, about 3-5% of the contigs had much longer lengths in the range of 1000 bp to 5000 bp and even for these long contigs there was no correlation between the contig length and maximum pA tract contig length (*r* = -0.05, P-value = 0.5, Sample size = 124) and they arise in almost equal proportions from the 5’ and 3’ introns of the transcript. In sum, the specific characteristic length distribution and selectivity of introns with cytoplasmically retained sequence seem to display distinct signatures of specific processing for CIRT biogenesis independent of the aRNA procedure. A visual representation for few randomly selected CIRTs has been created in UCSC Genome Browser and is provided in the supplementary (see Figure S4 in File S1).

**Figure 3:**
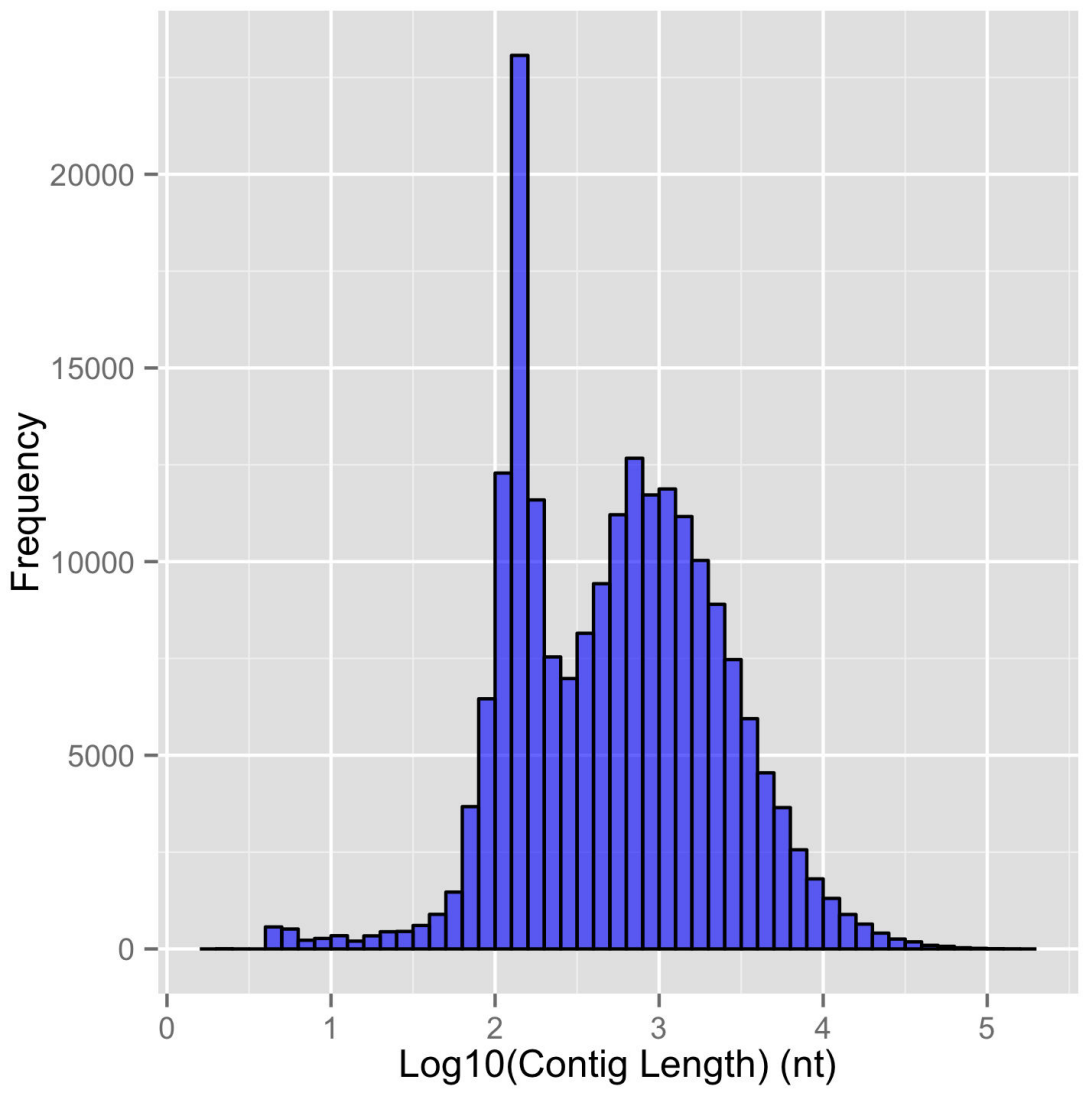
Contig length distribution for introns in total RNA from rat hippocampal tissue sample.

We also carried out total RNA sequencing from rat hippocampal tissue using Illumina NextGen sequencing and analyzed this data for the presence of intronic contigs. Intronic reads in tissue sample can arise as a result of the presence of incompletely spliced nuclear mRNAs as well as putative CIRTs. A large fraction of introns (~85%) from the transcripts present in the tissue sample were found to give rise to intronic contigs. However, the contig lengths were significantly longer (mean length ~ 1500 bp). (Figure 3). We found ~93% of the putative CIRTs in the rat dendrites to also have intronic reads in the rat tissue sample. The contig length of intronic sequences was significantly smaller in the dendritic CIRT sample as compared to the tissue sample (Wilcoxon test P value < 2.2e-16). Figure 3 also shows two modes for the tissue intronic contig lengths, one at ~300 bp and another at ~1000bp, suggesting the presence of a distinct shorter class of intronic sequences that are similar in length to hypothesized CIRTs. To rule out the possibility that this shorter class of intronic contigs are an artifact of retrotransposons, we analyzed for overlap with repeat families using RepeatMasker annotation available from UCSC genome browser database and after filtering out all the contigs that overlapped with any of the repeats we still continued to observe this mode at ~300 bp (of the 12,859 contigs in the length range 200-300, 7,386 (57.4%) contigs did not overlap by even a single base with any repeat). The tissue transcripts with the shorter ~300 nt intronic contigs also exhibit some other properties of the CIRTs. Like the putative CIRTs, only a small fraction of all the introns present in the genes of these transcripts (~30%) gave rise to the intronic contigs less than 300 nt in length. This is a much reduced fraction of introns considering that overall ~85% of all introns in tissue sample contained intronic contigs. Furthermore, the transcripts that comprised of intronic contigs <= 300 nt also exhibited high selectivity in terms of the number of introns from the transcript that gave rise to the <=300 nt contig (median number of introns with <=300 nt contig = 2, standard deviation = 3). This selectivity among the introns with short contig lengths suggests that these subset of transcripts represent the CIRTs. The sequencing data from tissue level samples is consistent with the hypothesis that the short intronic contigs forming the CIRTs in our dendritic samples show statistical signatures of specific lengths and location within multiple introns of a given gene. 

After having analyzed the properties of putative CIRTs from the carefully dissected dendrites that are free of nuclear hnRNA, we turned our attention to the whole single cell. Single cell transcriptomes are also expected to contain mostly cytoplasmic RNA (see 13) but since they do contain the nucleus we cannot completely rule out the presence of hnRNA. Following the pipeline outlined above we surveyed three individual single cell soma samples from rat hippocampal neurons and three individual single cell soma samples from mouse hippocampal neurons for CIRTs. We observed that the intronic contigs in the rat and mouse soma samples were longer than the contigs in rat and mouse dendritic samples, respectively (mean length of ~412 nt in rat soma samples and 392 nt in mouse soma samples, Wilcoxon test P-value < 2.1e-13) suggestive of the effect of hnRNA. Thus, to be conservative, we decided to retain only the intronic contigs that had a length of <= 400 nt. This results in a reduction of about one-third in the number of intronic contigs and about one-forth reduction in the total number of genes with intronic contigs. We observed ~44% of rat genes and ~47% of the mouse genes in soma samples with evidence of CIRTs (Table 1). Similar to the dendritic samples, we found that cytoplasmic retention occurs in only a small fraction of the introns (12-16%) (Table 2) in rat as well as mouse soma samples. These contigs from soma samples were also assessed as unlikely to code for proteins as per the criteria we used above for testing the dendritic contigs for protein coding potential. For the rat as well as mouse soma samples the pair-wise overlap among the genes that resulted in CIRTs was ~18% on average. For these shared genes the pair-wise overlap among the retained introns in these different cells was on average ~27%. The pair-wise overlap of all retained introns amongst the different cells ranged between 6-8% with a small fraction of ~2% of the introns appearing in CIRTs in all the samples.

To test the idea that CIRTs are a specific subset of a given intron’s sequences, we looked for read evidence of novel intron-intron junctions within a given intron. That is, evidence of editing of an intron different from annotated splicing patterns. We used the RUM pipeline [26] which is able to align reads across splice junctions *de novo*. By considering only the high quality junctions reported by RUM which have known splice signals and are crossed by at least one uniquely mapping read with at least 8 bp on each side of the junctions, we found several novel intron-intron junctions with canonical or known non-canonical splice signals in the both rat and mouse dendritic as well as soma samples (Table 3) (Table S3). In order to rule out the possibility that these novel intron-intron junctions arise due to contamination, we performed a cross-species mapping using NCBI Blast and the reads corresponding to the intron-intron junctions did not map to any other species.

**Table 3:** Novel intron-intron junctions present in the rat and mouse dendritic and soma samples.

	**#intron-intron junctions**	**#ID elements in the intron-intron junctions**
**Rat Dendrite**	869	81 (55 jns.)
**Rat Soma**	1076	104 (57 jns.)
**Mouse Dendrite**	2499	78 (47 jns.)
**Mouse Soma**	1012	6 (6 jns.)

In order to validate the presence of these junctions we selected mRNA from rat hippocampus tissue and carried out PCR amplification and sequencing using primers designed for intronic region surrounding the intron-intron junctions (see Methods). Additionally we also analyzed the genomic DNA from rat hippocampus to verify whether the putative spliced intronic region is in fact present in the genome. The rat strain used for obtaining the mRNA and genomic DNA was Sprague-Dawley which is the same strain from which we got the dendritic samples. Even then there is a potential for copy number variations between individual animals that cannot be ruled out. Of all the novel intron-intron junctions in rat samples, we selected the junctions for experimental validation based on the following criteria: i) junctions for which there was evidence for sufficiently long cytoplasmic retained intronic sequence surrounding the junction with high complexity sequences for primer design, ii) above median expression of the transcript in rat hippocampus tissue, iii) length of the intron containing the junction is shorter than 20,000 bp, and iv) junctions with spliced out length of >= 100 bp. This left us with 78 putative rat intron-intron junctions of which we selected 7 junctions that were detected in either rat soma or dendrites (Table S4). For two of the seven intron-intron junctions, the genomic DNA also showed a band corresponding to the spliced length which indicates that the observed splicing is likely due to SINE and other genomic insertions in the reference genome that are not present in the genome of the rat strain of our samples (Figure S5 in File S1, Lanes 2 and 3; Lanes 9 and 10). Initially, we observed unambiguous support for the intron-intron junction in only one of these junctions (Figure S5 in File S1, Lanes 16 and 17). For the remaining four junctions we did not find initial support for splicing (Figure S5 in File S1, Lanes 5 and 6; Lanes 12 and 13; Lanes 19 and 20; Lanes 23 and 24) which we hypothesized as likely due to the intron-intron junction being a minor variant form (File S1). In addition since we used tissue samples that contain a mixture of processed and unprocessed mRNAs the quantities may not have been sufficient for detection. For these junctions that could not be detected, we then designed primer pairs such that one primer spans the junction. Therefore, only the minor variant in the RNA pool that still contain the intron-intron junction may be specifically amplified. By doing so we found evidence for all four of the junctions which could not be validated previously (Figure S5 in File S1, Lanes 7, 14, 21 and 25 ). Thus five of the seven putative intron-intron junctions were secondarily validated as putative splice sites. Furthermore, all these PCR products have been sequence verified. Thus, presence of these intron-intron junctions supports the idea that CIRTs are the results of intron processing in the nucleus. 

Lastly, we also surveyed the EST database available on UCSC Genome Browser [27] to assess whether evidence of CIRTs previously existed in the EST libraries. We found that greater than 30% of the contigs in mouse dendrites as well as soma were completely contained within the EST sequences (Table 4). For rat dendritic and soma contigs this percentage was ~15-17% (Table 4). The smaller overlap for rats is most likely due to the less comprehensive EST data for the rat as compared to mouse. Thus, we believe that evidence for CIRTs existed in previous genomic data for expressed transcripts.

**Table 4:** Overlap between the retained intronic contigs and ESTs.

**Sample**	**# Contigs**	**# Contigs contained within ESTs**	**#ESTs within the contig**	**Partial overlap between contigs and ESTs**
**Mouse/Dendrite**	26,445	8,018 (30.3%)	72 (0.3%)	297 (1.1%)
**Rat/Dendrite**	8,459	1,343 (15.8%)	30 (0.3%)	125 (1.5%)
**Rat/Soma**	3,784	667 (17.6%)	2 (0.05%)	49 (1.3%)
**Mouse/Soma**	8,691	3,217 (37%)	8 (0.1%)	188 (2.2%)

### Non-neuronal cells also show evidence for CIRTs and suggests cell type specificity of CIRTs

Having observed a widespread presence of CIRTs in our neuronal samples, we were curious about their presence in other cells. To do so we analyzed some non-neuronal single cell datasets, namely five independently collected single cells from mouse brown adipose tissue (BAT) [14] and eight single cells from mouse cardiomyocytes (see Methods) using the same analysis pipeline for CIRTs. Since these samples are not mechanically isolated to be limited to the cytoplasmic fraction, we only retained the intronic contigs <= 400 nt to minimize any hnRNA contamination, as done above for soma samples. For these two cell types we also see a widespread prevalence of CIRTs (Figure S1 in File S1). In BAT cells we observed ~61% of the genes giving rise to CIRTs whereas in cardiomyocytes 49% of genes resulted in CIRTs. The pair-wise overlap among the genes that resulted in CIRTs between the five BAT single cells was ~23% on average. On average, when two BAT cells have retained intronic sequences in the same gene, in ~25% of the cases the retained sequence was from identical introns. Similarly, for the cardiomyocytes the pair-wise overlap among the genes that resulted in CIRTs between the eight single cells was ~22% on average. On average, when two cardiomyocyte cells have retained intronic sequences in the same gene, in ~29% of the cases the retained sequence was from identical introns. Thus here too we observe the cell to cell variability in the intronic reads similar to what we saw for the neuronal samples. 

For the whole single cell samples: mouse soma, BAT and cardiomyocyte samples, we found the average pair-wise overlap among the genes giving rise to CIRTs to be ~44%. On average, when two cells have retained intronic sequences in the same gene, in ~23% of the cases the retained sequence was from identical introns. A gene may show shared evidence of retained introns in various different cell types, but the particular retained introns may differ. For example, one such gene was the MLH3 gene which encodes the DNA mismatch repair protein Mlh3. This gene is associated with mammalian microsatellite instability [28]. This gene gave rise to CIRT with reads arising only from i10 (111 reads) in mouse soma, reads arising only from i8 (455 reads) in BAT cells, and reads arising only from i5 (277 reads) in cardiomyocyte cells. Such cases of CIRT switching between cell types are worth investigating further as it may indicate cell type dependent regulation of CIRT forms. 

### Genes with high intronic expression are enriched for GO categories involved in neuronal development

Given the widespread prevalence of CIRTs as detected by our analysis we investigated the functionality of the genes giving rise to CIRTs. It is possible that for majority of genes with CIRTs, the CIRTs comprise a minor transcript form while majority of the transcripts do not contain CIRTs as shown in [29]. These minor form CIRT may play a critical role in subcellularly regulated function or cell type specific function. But for the purpose of this analysis, we concentrated on the class of CIRTs where we had evidence that CIRTs comprise the major or plural form of transcript. In the previous section, we showed that even at a high intronic expression threshold (>=256 reads) there were abundant number of CIRTs (Figure S1 in File S1). We found these genes with greater than 256 intronic reads from at least one intron have high ratio of intron/exon read density ratio. In rats, we observed a median intron/exon ratio of 0.8 in the 256+ read class versus 0.1 for all other CIRTs and in mice we observed a median intron/exon ratio of 2.3 versus 0.4 for all other CIRTs. Hence we focused on this set of genes to perform a GO analysis. For all GO enrichment analysis described below the background set was comprised of the genes expressed in the sample being investigated. 

 There were 707 genes in rat dendritic samples with intronic read count >= 256 from at least one retained intron. We performed GO analysis on this set using DAVID Bioinformatics Resources 6.7 [30,31]. Among the GO terms that were significantly enriched, there were GO terms involved in important functionalities in the neuron such as neuron development, neuron projection development, cell projection organization and cellular component morphogenesis (P-value < 4.23E-05, Benjamini p-value < 0.006). The biological processes involved in protein localization were also significantly enriched (P-value < 1.08E-05, Benjamini p-value < 0.003). Included in the genes with enriched GO terms were several genes that have been implicated in neuronal diseases. For instance, it includes the Huntingtin gene which is especially interesting in context of neurodegenerative diseases. Atlastin-1 is involved in spastic paraplegia which is a cognitive and motor disease [32]. Reelin has been implicated in schizophrenia [33].  Ataxin-10 is involved in neurodegeneration and maybe associated with neurodegenerative diseases [34]. In mouse dendrite samples there were 2,021 genes >= 256 read counts from at least one intron. Here among the significantly enriched terms we found biological processes such as cell projection organization (P-value < 2.28E-04, Benjamini p-value < 0.02) and protein localization (P-value < 5.84E-05, Benjamini p-value <0.04). Similarly, we carried out GO analysis for genes with high intronic read count from BAT and cardiomyocyte single cells. In BAT cells there were 2,341 genes having >= 256 reads from at least one intron. We found the GO term corresponding to biological adhesion and cell adhesion significantly enriched (P-value = 3.91E-07, Benjamini p-value = 0.001). In cardiomyocytes there are 1,326 genes with >= 256 reads from at least one intron. Here, we found the GO terms corresponding to heart development (P-value = 9.2E-05, Benjamini p-value = 0.02) and KEGG pathways corresponding to dilated cardiomyopathy (P-value = 8.08E-06,Benjamini p-value = 6.7E-04), arrhythmogenic right ventricular cardiomyopathy (P-value = 2.01E-05, Benjamini p-value = 0.001) and hypertrophic cardiomyopathy (P-value = 8.6E-05, Benjamini p-value = 0.003) significantly enriched. We emphasize that the background for these enrichment tests were the genes expressed in each cell type, thus, these results suggest that genes involved in neuronal function and heart function are especially prone to high degree of intron retention, above and beyond standard expression of their cell-specific transcriptome. Table S5 lists all of the enriched GO terms in all of the samples along with the genes involved.

### ncRNAs within CIRTs were predominantly involved in splicing

As reported in the above, as part of our filtering step we analyzed for overlap of CIRTs with ncRNAs. We found <2% of the retained introns in mouse and rat dendrite samples to have the Rfam [21] annotated RNA families and RNA genes embedded within them. Although, we did filter out these introns from our list of CIRTs, we were curious about which ncRNAs they contained. Interestingly, of the functional ncRNAs that we found to be embedded, ~30% (Chi-squared test p-value = 0.01) were members of small nuclear RNAs (snRNAs) that make up the major and minor spliceosome (U1, U2, U4, U5, U6, U11, U12, U6atac), and U7 snRNA which is involved in the splicing of metazoan histone pre-mRNAs [35,36]. It has been reported that the minor spliceosome snRNAs (U11, U12, U4atac, and U6atac; as well as U5 snRNA which is shared with the major spliceosome) are predominantly cytoplasmic in vertebrates and are responsible for the splicing of partially spliced pre-mRNAs containing minor-class introns that have been exported from the nucleus [37]. Curiously, also among these embedded ncRNAs was the 5S ribosomal RNA (5S rRNA) (comprising ~5% of all ncRNAs mapping to CIRTs) that makes up the large subunit of the ribosomal RNA. Another ncRNA that we found was the 7SK small nuclear RNA (snRNA) (comprising ~10% of all ncRNAs mapping to CIRTs) which has been shown to act as a negative regulator of the RNA polymerase II elongation factor P-TEFb [38]. In addition ~10% of all ncRNAs that mapped to CIRTs were miRNAs. To further analyze the possibility that functional ncRNAs may be co-transcribed as part of the CIRTs rather than an independent transcript, we looked for evidence such as intron-exon junction reads extending the intronic reads which may suggest that the ncRNAs are co-transcribed with the expressed genes and exported as CIRTs. We found 11 instances of rat intronic contigs and 7 instances of mouse intronic contigs with evidence for intron-exon junction reads and the intronic contig overlapping with functional ncRNAs. Ten of these 11 rat contigs overlapped the small nucleolar RNAs (snoRNAs) [39] and one overlapped with the 7SK snRNA. Five of the 7 mouse contigs overlapped with the snoRNAs as well and the other two overlapped U1 and U4 snRNAs.

## Discussion

In this paper, we use RNA sequencing data from micro-dissected cytoplasmic compartments to show evidence supporting genome-wide prevalence of cytoplasmic intron-sequence retaining transcripts (CIRTs). Surprisingly, up to 59% of expressed genes in rodent neurons showed evidence of sequence reads from intronic sequences. Similar proportions were also seen in mouse Brown Adipose Tissue (BAT) cells and mouse cardiomyocytes. Support for this genome-wide presence of CIRTs come from several sources. First, our RNA populations for the neuronal cells were derived from carefully dissected non-nuclear dendritic segments with little potential for contamination from hnRNA. This fact was supported also by distinct distribution of intronic RNA sequences in contrast to the distributions seen from single cell soma sequencing and whole tissue sequencing. Second, we used multiple computational filters to remove potential effects of cryptic exons, ncRNA, etc. Third, previous work from our lab showed extensive evidence for CIRTs using PCR cloning, in situ hybridization, and RNA sequencing leading to the hypothesis that CIRTs were prevalent across the transcriptome [13]. In addition, existing datasets such as EST databases show broad evidence of CIRTs that previously were not interpreted as cytoplasmic intronic sequences. Finally, accumulating literature suggests cytoplasmic presence of intronic sequences is an important part of the repertoire of post-transcriptional regulation [6,10,29,40,41]. Recent introduction of RNA sequencing has led to the realization that much greater proportion of the genome is transcribed [42] and that the transcriptome is much more diverse and complex than previous models. Many of the RNA sequencing datasets have shown high levels of intronic sequences (per. comm.). In fact, as shown above, existing EST datasets also show evidence of intronic sequences. Previously these sequences were interpreted as hnRNA but here we suggest that specific subsequences of introns are retained for post-transcriptional processes and that the extent of such intron retention is genome-wide.

If CIRTs play a role in post-transcriptional cell physiological processes, we hypothesize that non-random subsequences of introns are specifically retained rather than retaining any introns of a given gene. Many mammalian introns are extremely large and the median size of mouse and rat introns is ~1300 bp (standard deviation = ~18,000 bp). The contigs assembled from our RNA sequencing suggest typical CIRT size in the range of 350-400 bp, suggesting that the CIRT region of a transcript is processed in the nucleus to a specific subsequence. This size range data is supported by existence of a sharp second mode around 300 bp in intronic sequences from normal tissue RNA sample. In addition, when we find evidence of cytoplasmic intronic sequences for a given gene, typically the sequence evidence is present for only a specific intron rather than all of the gene’s introns. Finally, we found numerous examples of intron-intron junctions surrounding the CIRTs and carried out experiment validation for seven putative intron-intron junctions. We were able to show evidence of spliced product for five of these junctions. We note that our annotation of the intron-intron junctions were limited to known canonical and non-canonical splice signals and if we were to expand the definition to novel junction types, we may have considerably larger annotation of intron-intron processing. Thus from these data we hypothesize that at least some proportion of transcripts for many genes in the genome are alternatively processed during splicing to retain specific subsequences of intronic sequences. Recently, there have been other findings that also lead to the speculation of alternate splicing mechanisms giving rise to a diversity of transcripts not thought to exist previously. For instance, the detection of chimeric RNAs comprising of sequences from different genic boundaries, even those that are on different chromosomes, suggest the involvement of an alternate splicing machinery [3]. 

The functional roles of CIRTs are uncertain at this stage. In Buckley et al. [13], we demonstrated that CIRTs can contain sub-families of SINE elements from the ID family, which confers dendritic localization. Other known case studies suggest regulation of translation efficiency [40], alternate protein isoforms in response to external stimulus [41] and splice variant diversity [29]. We noted above that genes with high intronic sequence retention in neurons as well as BAT and cardiomyocyte cells tend to be enriched for processes involved in protein and mRNA transport and localization along with other cell type specific functions. Neurons and other cells in the CNS are arguably some of the most complex cell types and CIRTs may provide yet another level of regulation by which complexity of molecular physiology is generated. One speculative function of CIRTs is that they may carry functional subsequences that either play a role prior to translation (e.g., ID elements for localization) or play a role after cytoplasmic splicing and release from the coding transcript. We noted that some of our CIRTs overlap with Rfam annotated ncRNAs. Specifically, a large fraction of these ncRNAs were members of snRNAs that make up the major and minor spliceosome, 5S rRNAs, snoRNAs, miRNAs, and other small RNAs. Cytoplasmic splicing and release of these functional subsequences may provide signals related to translational processing of specific mRNA. 

In conclusion, we hypothesize that retention of subsequences of introns involves specific alternative splicing pathways and such sequence retention is wide spread for genes in the mammalian genome. We speculate that these CIRTs play a role in post-transcriptional regulation and possible RNA-based signaling after cytoplasmic processing. Overall, the presence of CIRTs increases the complexity of the transcriptome and CIRT modulation may present another pathway for epigenesis of cellular complexity and play a role in neuronal plasticity.

## Methods

### Culturing Conditions

Hippocampi were harvested from embryonic day 18 rat (Sprague-Dawley) and mouse (C57BL/6) pups and dispersed and plated at 100,000 cells per ml of neurobasal medium and B27 (Invitrogen). Neurons are grown in culture for 14 days on 12 mm round German Spiegelglas coverslips (Bellco) coated with poly-L lysine (Peptide Institute).

### mRNA Amplification

Independent samples of two sets of approximately 150-300 dendrites and 3 cell soma from primary rat hippocampal neurons and three sets of approximately 150-300 dendrites and 3 cell soma from mouse hippocampal neurons were harvested by mechanical isolation and used as template material for aRNA amplification (as described in [25]).

### Illumina Sequencing

Paired-end libraries were constructed from fragmented dendritic and soma aRNA material and sequenced by using Illumina II short-read technology. Paired-end libraries were also constructed for total RNA from whole hippocampus tissue and sequenced using Illumina II short-read technology. The read length is 50 bp. Reads were trimmed for adapter sequence and poly-A, poly-T and then aligned to the Rat (rn4) and Mouse (mm9) genome by using the RUM pipeline [26]. The RUM pipeline also reports *de novo* junctions. The number of reads in each library and alignment statistics are provided in Table S6. All sequence data for this project has been deposited at NCBI GEO database under accession number GSE49592.

### BAT and cardiomyocyte single cells

The BAT cells were collected in the lab of J. Eberwine [14]. Briefly, Animals Interscapular brown adipose tissue was isolated from E17.5 CD-1 mice and subjected to collagenase digestion, fractionated and isolated mature brown adipocytes plated on glass coverslips in 35-mm plates. The BAT cells identified using morphology and were harvested by mechanical isolation. 

The cardiomyocytes were collected in the lab of B. Kuhn. Briefly, embryonic cardiomyocytes were isolated from the ventricles of 14.5 day old of a hemizygous Fucci (Fluorescent Ubiquitination-based Cell Cycle Indicator) embryo [43]. The cardiomyocytes were disassociated using a modified protocol of the commercially available cardiomyocyte isolation kit (Neomyts kit, Cellutron Life Technologies). Freshly isolated cells were flow sorted on a FACSAria (Becton Dickenson Biosciences) instrument for fluorescence and viability. 

RNA from these single cells were collected, amplified and sequenced as described above.

### Animal Protocols

The collection of the primary cultured cells utilized animal by-products protocol, "Genome Biology of Single Neuron Funciton and its Modulation with Age" under University of Pennsylvania IACUC protocol #803321 (Sept. 22, 2010 approval). Animals were sacrified under University of Pennsylavania IACUC protocol #804867, "Molecular Biology of Single Aging Neurons and Glia" (May 15, 2013 renewal), but the sacrifice of the animals were independent of the work reported in this paper. The cardiomyocytes were collected under Harvard University IACUC protocol 12-05-2169R, "Mechanisms of myocardial regeneration" (approval 5/23/2012). All protocols were approved by University of Pennsylvania and Harvard Office of Regulatory Affairs and IACUC committee.

### Novel intron-intron junction validation

Primers were designed from the intronic region surrounding the putative intron-intron junction for which we had sequence evidence for cytoplasmic retention (Table S7). cDNA was generated from rat hippocampal mRNA isolated from dissected whole tissue. Additional cDNA was generated from a commercial source of rat hippocampal ss cDNA (Zyagen, San Diego, CA). Genomic DNA (gDNA) was extracted from rat hippocampal tissue from the same strain as the dendritic mRNA. The gDNA was isolated to a concentration of 82 ng/uL. 1 uL of the gDNA was used in each positive control PCR. PCR conditions were optimized for the highest specificity for each individual junction. The PCRs were run at the following conditions on cDNA with primers surrounding the putative intron-intron junction: NM_001100548, NM_001107789, NM_001106758, NM_017216, NM_001108997 and NM_001047860: 94 °C for 2 min (x1), then for 40 cycles; 94 °C for 30 sec; 59 °C for 1 min; 72 °C for 1 min. NM_001047925: 94 °C for 2 min (x1), then for 40 cycles; 94 °C for 30 sec; 57 °C for 1 min; 72 °C for 1 min. The PCRs were run at the following conditions on gDNA with primers surrounding the putative intron-intron junction: NM_001100548, NM_001107789, NM_001106758, NM_001108997 and NM_017216: 94 °C for 2 min (x1), then for 40 cycles; 94 °C for 30 sec; 59 °C for 1 min; 72 °C for 1 min. NM_001047860: 94 °C for 2 min (x1), then for 40 cycles; 94 °C for 30 sec; 61 °C for 1 min; 72 °C for 1 min. NM_001047925: 94 °C for 2 min (x1), then for 40 cycles; 94 °C for 30 sec; 57 °C for 1 min; 72 °C for 1 min. The PCRs were run at the following conditions on cDNA with junction-spanning primer: NM_001108997: 94 °C for 2 min (x1), then for 40 cycles; 94 °C for 30 sec; 57 °C for 1 min; 72 °C for 1 min. NM_001100548, NM_001107789 and NM_017216: 94 °C for 2 min (x1), then for 40 cycles; 94 °C for 30 sec; 59 °C for 1 min; 72 °C for 1 min. All PCR programs ended at 4 °C. The completed reactions were run on a 2% agarose & ethidium bromide (EtBr) gel at 115 volts and imaged with UV light. The completed reactions were run on a 2% agarose & ethidium bromide (EtBr) gel at 115 volts and imaged with UV light. 

### Background Intron Set

For every intron in the dendritic samples that showed evidence for cytoplasmic intron sequence retention, we selected another intron randomly such that it had a similar length and GC content (within a range of 5%) as well as an identical internal pA-tract length and belonged to a gene that had no cytoplasmic intron sequence retention. In this way we were able to construct a background intron set size matched with the set of retained introns for both mouse as well as rat. 

## Supporting Information

File S1 Figure S1, Fraction of CIRTs as a function of reads mapping to the retained intronic contigs. Figure S2, Fraction of retained introns/gene as a function of total number of intronic reads/gene. Figure S3, Fraction of intronic contigs falling within each one-third region of the introns. Figure S4. Visual representation of the reads mapping to randomly picked genes that give rise to putative CIRTs. Figure S5, Experimental validation by RT-PCR of seven putative intron-intron junctions from rat dendrites and soma. Table S1, Position of Rat and Mouse dendrites retained introns within the gene (for genes with at least 5 introns). Table S2, Retained introns in Rat and Mouse dendrites with internal pA-tract of varying lengths.
(DOC)

Table S3 **List of novel intron-intron splice junctions.**
(XLS)

Table S4 **Experimentally validated intron-intron splice junctions.**
(XLS)

Table S5 **Gene ontology analysis results.**
(XLSX)

Table S6 **Next generation sequencing alignment statistics.**
(XLS)

Table S7 **List of primers used for experimental validations.**
(XLS)
