# Supplementary material for: Subcellular RNA Sequencing Reveals Broad Presence of Cytoplasmic Intron-Sequence Retaining Transcripts in Mouse and Rat Neurons
Source: PLoS One. 2013 Oct 3;8(10):e76194. doi: 10.1371/journal.pone.0076194 (PMC3789819; doi:10.1371/journal.pone.0076194)
Supplement: File S1 — Figure S1, Fraction of CIRTs as a function of reads mapping to the retained intronic contigs. Figure S2, Fraction of retained introns/gene as a function of total number of intronic reads/gene. Figure S3, Fraction of intronic contigs falling within each one-third region of the introns. Figure S4. Visual representation of the reads mapping to randomly picked genes that give rise to putative CIRTs. Figure S5, Experimental validation by RT-PCR of seven putative intron-intron junctions from rat dendrites and soma. Table S1, Position of Rat and Mouse dendrites retained introns within the gene (for genes with at least 5 introns). Table S2, Retained introns in Rat and Mouse dendrites with internal pA-tract of varying lengths. (DOC) [file pone.0076194.s001.doc]

**Supplementary Text**

**Novel intron-intron junctions**

The average coverage for the novel intron-intron junctions that we observed to be present in the CIRTs was ~0.05 reads/bp which is about one tenth of the average coverage in the intronic contigs reported above. However, for ~40% of these junctions this low coverage is explained by reads not in support of the junction indicating that the intron-intron junction may be a minor variant form.

**Supplementary Figures**

**
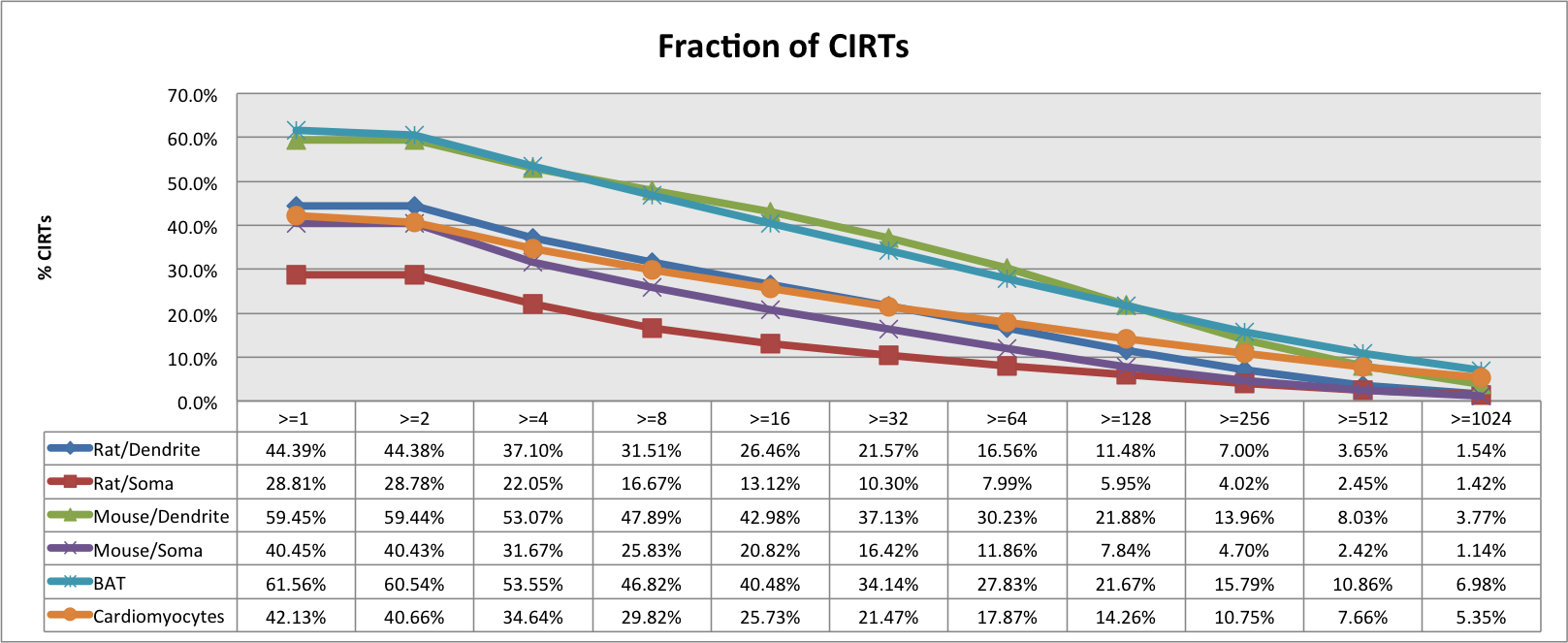
**

**Figure S1.** Fraction of CIRTs as a function of reads mapping to the retained intronic contigs


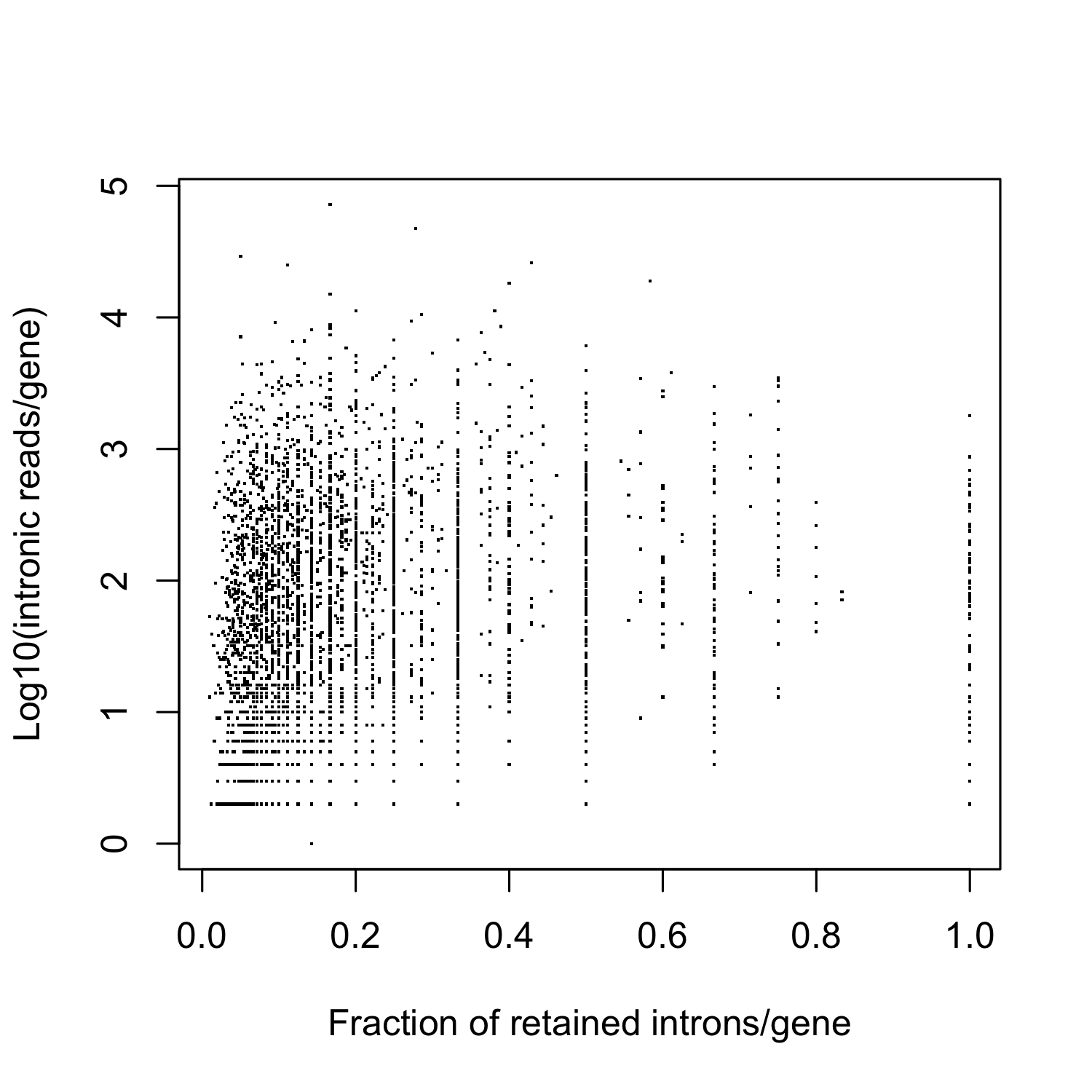


**Figure S2.** Fraction of retained introns/gene as a function of total number of intronic reads/gene


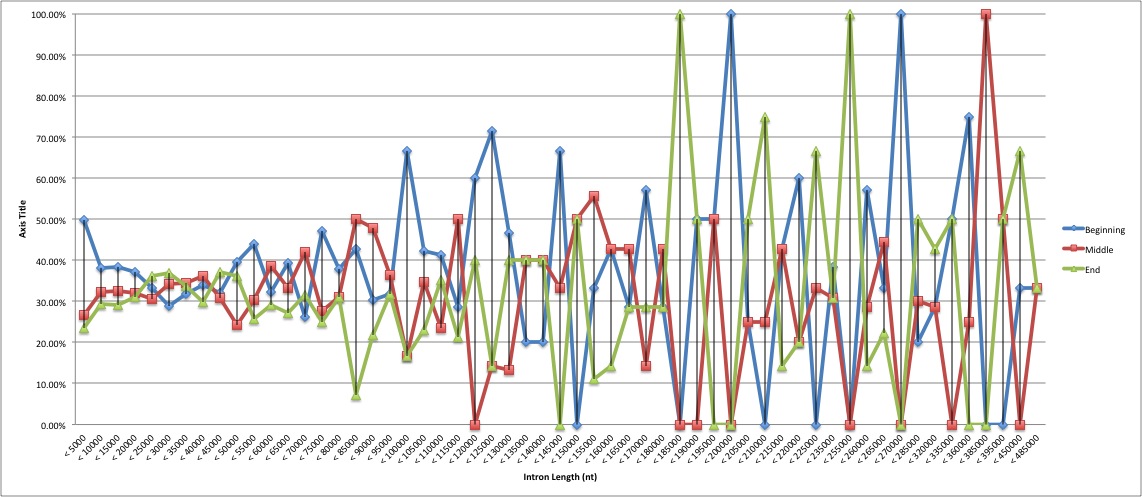


**Figure S3.** Fraction of intronic contigs falling within each one-third region of the introns


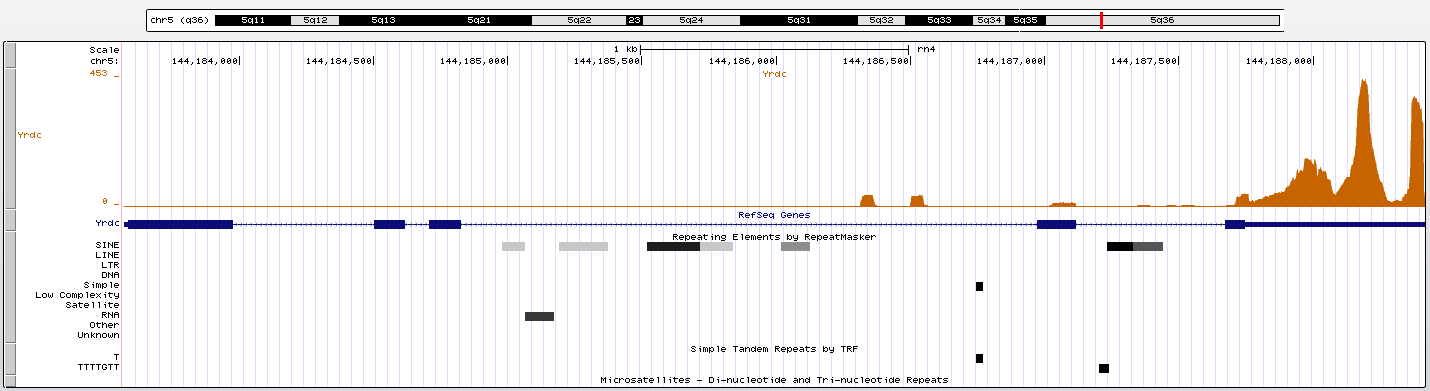


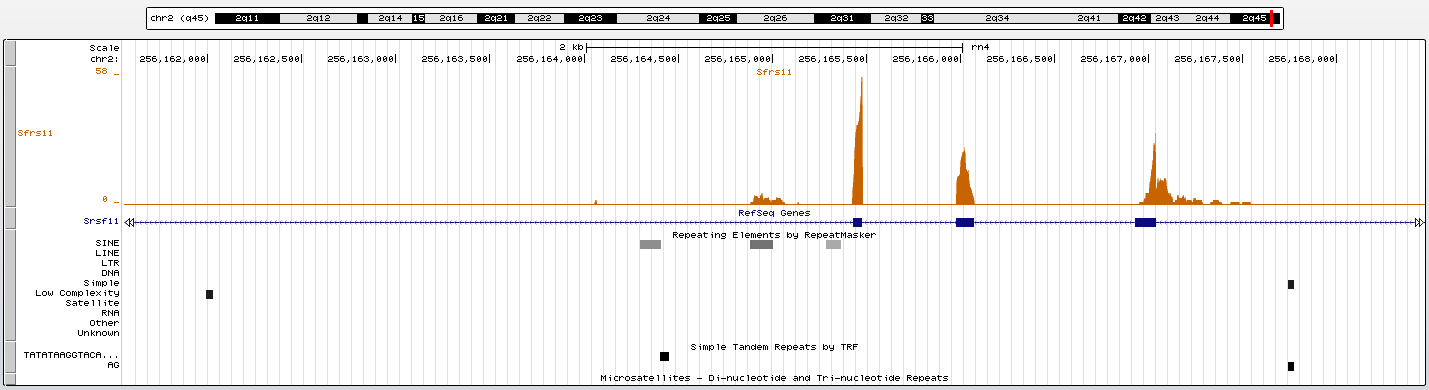


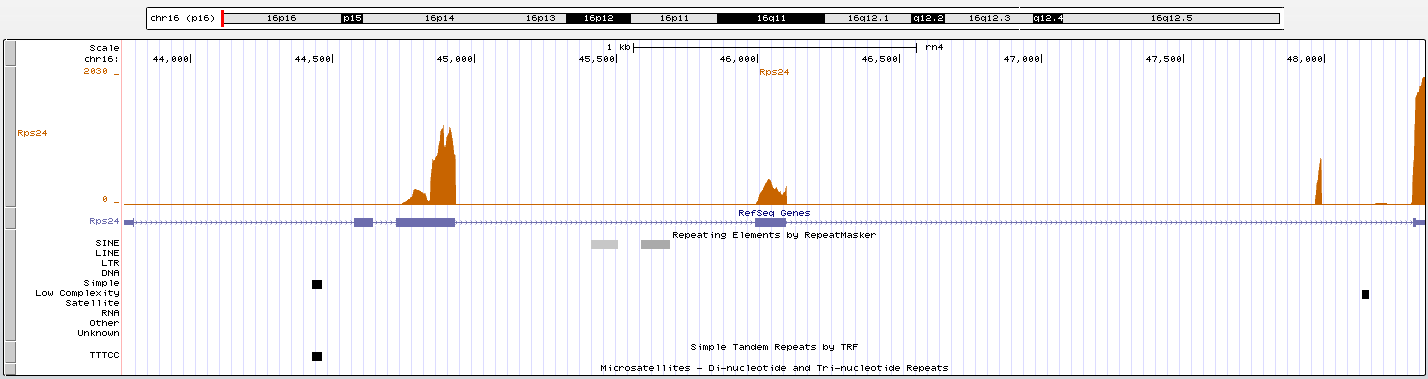


**Figure S4.** Visual representation of the reads mapping to randomly picked genes that give rise to putative CIRTs


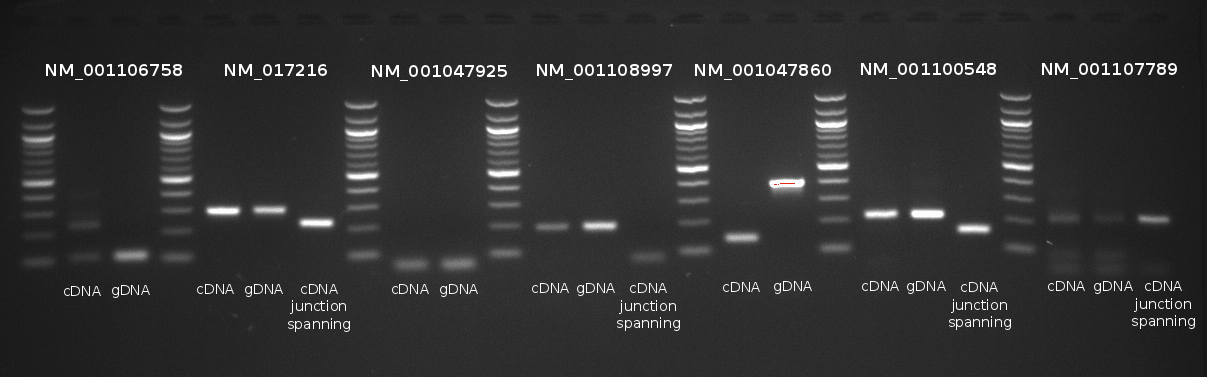


**Figure S5.** Experimental validation by RT-PCR of seven putative intron-intron junctions from rat dendrites and soma. The products in the lanes are as follows: Lane 2: NM_001106758 cDNA with primers designed from around the intron-intron junction; Lane 3: NM_001106758 gDNA with primers designed from around the intron-intron junction; Lane 5: NM_017216 cDNA with primers designed from around the intron-intron junction; Lane 6: NM_017216 gDNA with primers designed from around the intron-intron junction; Lane 7: NM_017216 cDNA with left primer designed to span the intron-intron junction; Lane 9: NM_001047925 cDNA with primers designed from around the intron-intron junction; Lane 10: NM_001047925 gDNA with primers designed from around the intron-intron junction; Lane 12: NM_001108997 cDNA with primers designed from around the intron-intron junction; Lane 13: NM_001108997 gDNA with primers designed from around the intron-intron junction; Lane 14: NM_001108997 cDNA with left primer designed to span the intron-intron junction; Lane 16: NM_001047860 cDNA with primers designed from around the intron-intron junction; Lane 17: NM_001047860 gDNA with primers designed from around the intron-intron junction; Lane 19: NM_001100548 cDNA with primers designed from around the intron-intron junction; Lane 20: NM_001100548 gDNA with primers designed from around the intron-intron junction; Lane 21: NM_001100548 cDNA with left primer designed to span the intron-intron junction; Lane 23: NM_001107789 cDNA with primers designed from around the intron-intron junction; Lane 24: NM_001107789 gDNA with primers designed from around the intron-intron junction; Lane 25: NM_001107789 cDNA with left primer designed to span the intron-intron junction

**Supplementary Tables**

**Table S1. Position of Rat and Mouse dendrites retained introns within the gene (for genes with at least 5 introns)**

|  | **# Introns** | **# Retained introns** | **# Retained introns from 5’ half** | **# Retained introns from 3’ half** |
| --- | --- | --- | --- | --- |
| **Rat** | 54,996 | 6,495 | 3,012 (46.4%) | 3,483 (53.6%) |
| **Mouse** | 118,147 | 17,946 | 9,527 (53.1%) | 8,419 (46.9%) |

**Table S2. Retained introns in Rat and Mouse dendrites with internal pA-tract of varying lengths**

|  | **#Retained introns** | **# Retained introns with internal pA tract** | | | | | |
| --- | --- | --- | --- | --- | --- | --- | --- |
|  |  | **>= 11 bp** | **>= 10 bp** | **>= 9 bp** | **>= 8 bp** | **>= 7bp** | **>= 6bp** |
| **Rat** | 7,426 | 3,112 (41.9%) | 3,522 (47.4%) | 3,947 (53.2%) | 4,381 (58.9%) | 4,994 (67.3%) | 5,651 (76.1%) |
| **Mouse** | 19,477 | 7,779 (39.9%) | 9,188 (47.2%) | 10,637 (54.6%) | 12,200 (62.6%) | 14,449 (74.2%) | 16,492 (84.6%) |
